# A carotenogenic mini-pathway introduced into white corn does not affect development or agronomic performance

**DOI:** 10.1038/srep38288

**Published:** 2016-12-06

**Authors:** Daniela Zanga, Teresa Capell, Gustavo A. Slafer, Paul Christou, Roxana Savin

**Affiliations:** 1 Crop and Forestry Sciences, School of Agrifood and Forestry Science and Engineering (ETSEA), University of Lleida-Agrotecnio Center, Lleida, Spain; 2 ICREA, Catalan Institute for Research and Advanced Studies, Passeig Lluís Companys 23, 08010 Barcelona, Spain

## Abstract

High-carotenoid corn (Carolight®) has been developed as a vehicle to deliver pro-vitamin A in the diet and thus address vitamin A deficiency in at-risk populations in developing countries. Like any other novel crop, the performance of Carolight® must be tested in different environments to ensure that optimal yields and productivity are maintained, particularly in this case to ensure that the engineered metabolic pathway does not attract a yield penalty. Here we compared the performance of Carolight® with its near isogenic white corn inbred parental line under greenhouse and field conditions, and monitored the stability of the introduced trait. We found that Carolight® was indistinguishable from its near isogenic line in terms of agronomic performance, particularly grain yield and its main components. We also established experimentally that the functionality of the introduced trait was indistinguishable when plants were grown in a controlled environment or in the field. Such thorough characterization under different agronomic conditions is rarely performed even for first-generation traits such as herbicide tolerance and pest resistance, and certainly not for complex second-generation traits such as the metabolic remodeling in the Carolight® variety. Our results therefore indicate that Carolight® can now be incorporated into breeding lines to generate hybrids with locally adapted varieties for further product development and assessment.

Carotenoids are essential pigments found mainly in plants and microbes^1^. They consist of eight isoprenoid units joined end to end to form a C_40_ hydrocarbon skeleton that includes a chromophore and linear or cyclic end groups. Plant carotenoids accumulate in plastids and play fundamental roles in photosynthesis as accessory pigments and in the xanthophyll cycle by preventing photo-oxidative damage^2^. They also facilitate plant interactions with the biotic environment by conferring characteristic colors to fruits and flowers, making them attractive to insects and thus favoring pollination and seed dispersal^1^. The degradation of certain carotenoids also provides precursors for the synthesis of abscisic acid (ABA)^3^,^4^. From a nutritional perspective, carotenoids such as β-carotene possess pro-vitamin A activity and are therefore essential for animals (including humans) that cannot synthesize retinoids *de novo*. Furthermore, carotenoids such as lycopene provide general health-promoting antioxidant activity whereas lutein and zeaxanthin specifically help to protect the retinal cells of the macula against phototoxic damage, thus preventing age-related macular degeneration (ARMD)^5^.

Several transgenic crops with high carotenoid levels have been generated by overexpressing carotenogenic genes, including Golden Rice II^6^ and high-carotenoid Carolight® corn^7^, both of which overexpress corn *psy1* and *Pantoea ananatis crt*I. Golden Rice II produces 37 μg g^−1^ dry weight (DW) of total carotenoids (26-fold more than the near isogenic wild-type variety) and Carolight® corn produces 84 μg g^−1^ DW of total carotenoids (156-fold more than the near isogenic wild-type variety)^6^,^7^. Such biofortified crops provide an excellent and sustainable means to combat vitamin A deficiency, which is prevalent in many developing countries^8^. Transgenic crops have increased the productivity and sustainability of agriculture, not only in industrialized countries but also in developing countries that have adopted them successfully^8^,^9^,^10^. As part of the development process, novel plant varieties including genetically engineered crops are tested under field conditions to determine whether their performance meets the standards set by commercial developers. The performance of novel crops (genetically engineered or otherwise) under laboratory and/or greenhouse conditions may differ considerably in the field, particularly metabolic or development traits, because the resources needed to produce novel heterologous molecules may compete with other processes, resulting in a trade-off between improved nutritional value and yield or agronomic performance. Both conventional breeding and genetic engineering may therefore affect physiological traits such as yield or biomass production in a positive^11^, negative^12^,^13^ or neutral manner^14^. A recent comprehensive transcriptomic, proteomic and metabolomic analysis revealed that the endosperm-specific carotenoid biosynthesis in Carolight® correlated with changes in endosperm carbohydrate metabolism, as well as sterol and fatty acid biosynthesis^15^. It is therefore important to test the performance of Carolight® under a range of conditions to ensure that the remodeled metabolic network does not cause a trade off with other beneficial traits, such as yield components. It is rare for even first-generation traits to be comprehensively tested against their near-isogenic wild-type counterparts under diverse conditions and unprecedented for complex second-generation traits involving metabolic engineering.

We therefore tested the hypothesis that Carolight® is similar in terms of agronomic performance to its non-transgenic near isogenic counterpart when compared in a controlled environment and under field conditions. The key objectives of our studies were (i) to determine whether the accumulation of carotenoids in Carolight® affects the rates of photosynthesis, biomass accumulation and partitioning into different organs in experimental field trials (study 1); and (ii) to compare the effectiveness of the introduced trait in a controlled greenhouse environment and the field (study 2). In order to broaden the range of environments and to test the consistency of adaptation and performance between Carolight® and its near isogenic counterpart, we compared both genotypes in field plots under different soil nitrogen (N) availability regimes and contrasting source-sink relationships during grain filing. N availability is known to influence photosynthesis and biomass accumulation^16^ and modifying the source-sink relationship may alter the grain weight and N concentration in the grain to differing degrees in different crops^17^,^18^. Such comprehensive testing is rare but necessary to assure the long term success of crops with novel traits when they are commercially released in different environments.

## Materials and Methods

### Treatments

We used two corn (*Zea mays*) near-isogenic genotypes: the wild-type white endosperm variety M37W and the high-carotenoid transgenic line Carolight®, expressing corn *psy1* and *Pantoea annatis crt*I in a M37W genetic background. M37W seeds were obtained from CSIR (Pretoria, South Africa). Seeds were planted in the experimental fields of the School of Agronomy, University of Lleida, Spain (41°37′50″N, 0°35′27″E, 180 m) (study 1) or in pots maintained in the greenhouse (study 2).

Two different fertilization regimes were applied: N_0_ = 0 kg ha^−1^ and N_200_ = 200 kg ha^−1^. The N was applied as urea at the V_6_ stage (six fully-expanded leaves). Source–sink relationships were manipulated at silking (the R_1_ stage) by removing most of the leaves, and tests were carried out using plants that were distributed uniformly at the same density and visually identical in terms of size, leaf number and developmental stage. Most of the leaf laminae were removed by cutting the leaves on the collar, between the lamina and sheath. Only the two leaves adjacent to the ear were left on the plant (those immediately below and above). The effects of all treatments and their interactions were tested for statistical significance by the analysis of variance (ANOVA).

### Experimental design

In study 1, seeds were sown in the field on 5 May 2013 and treatments consisted of a factorial combination of the two maize genotypes and two N treatments (N_0_ and N_200_) with subplots consisting of source–sink treatments. Plots were randomized with four replicates, each consisting of six rows, 70 cm apart and 6.47 m in length. Each plot was fully irrigated. Pests, diseases and weeds were controlled or avoided by spraying recommended insecticides, fungicides and herbicides at the doses suggested by their manufacturers whenever necessary.

In study 2, seeds were sown in 20-L pots in the greenhouse on 5 May 2013. For the first 50 days, the day/night temperature was 28/20 °C with a 10-h photoperiod and 60–90% relative humidity, and thereafter the day/night temperature was 21/18 °C with a 16-h photoperiod. Seeds were harvested at 15, 20, 25, 30, 40, and 60 days after sowing (DAS), and the dissected endosperm was frozen in liquid nitrogen and stored at −80 °C.

### Sampling and analysis of field plants

Three developmental stages were chosen for biomass sampling: V_8_ (eight fully-expanded leaves), R_1_ (silking) and R_6_ (maturity)^19^. Plants were cut at ground level, and the stems (including leaf sheaths), leaf laminae, ears and grains (at maturity) were separated in the laboratory. The ears were divided into basal, central and apical sections, and grains in each section were separated from the cob and counted. The area of green leaf tissue in all samples was determined at stages V_8_ and R_1_ using a Li-3100C area meter (Li-COR Biosciences, Lincoln, NE, USA). All plant materials were oven dried for 72 h at 65 °C after processing and weighed to determine the dry biomass of each fraction.

After weighing, all plant materials were ground in analytical mills, and the N concentration was determined using the Kjeldahl method. The specific leaf area (SLA) was calculated by dividing the leaf area per plant by leaf mass, and the specific leaf nitrogen (SLN) content was calculated as the ratio of leaf nitrogen content to leaf area. The leaf area index (LAI) was calculated as the total green area of leaf tissue per unit ground area. N utilization efficiency (NUtE) was calculated as the ratio between yield and N uptake at physiological maturity. The N harvest index (NHI) was calculated as the ratio between N content in the grains and aboveground biomass at maturity.

Photosynthetic rates and related traits were determined for individual leaves every 2 weeks from V_8_ until grain filling was complete, using a LCi portable photosynthesis system (ADC BioScientific, Great Amwell, UK) which measures net CO_2_ assimilation (net photosynthetic rate). Measurements were taken using the last fully-expanded leaf between mid-morning and noon on cloudless days by holding the photosynthetic chamber perpendicular to the direction of incident solar radiation. The leaf chlorophyll concentration from V_8_ to R_6_ was estimated *in situ* using a SPAD-520 portable chlorophyll meter (Minolta, Tokyo, Japan) on the same leaves used to measure gas exchange. The duration of photosynthesis was calculated by integrating values across the growth cycle and measuring the area underneath the curve^20^.

### Total RNA isolation and cDNA synthesis

Total RNA was isolated using the RNeasy Plant Mini Kit (Qiagen, Valencia, CA, USA) and DNA was removed with DNase I (RNase-free DNase Set, Qiagen). Total RNA was quantified using a Nanodrop 1000 spectrophotometer (Thermo Fisher Scientific, Vernon Hills, IL, USA), and 2 μg total RNA was used as the template for first strand cDNA synthesis with Ominiscript reverse transcriptase (Qiagen) in a 20-μl total reaction volume, following the manufacturer’s recommendations.

### Quantitative real-time RT-PCR

Quantitative real-time RT-PCR was used to analyze *Zmbch1*, *Zmbch2*, *Zmcrtiso, Zmcyp97a*, *Zmcyp97b*, *Zmcyp97c*, *Zmlycb*, *Zmlyce*, *Zmpds*, *Zmzds*, *Pacrt*I and *Zmpsy1* gene expression in a BioRad CFX96 system with 25-μl reaction mixtures containing 10 ng cDNA, 1x iQ SYBR Green Supermix (BioRad, Hercules, CA, USA) and 0.2 μM forward and reverse primers^21^,^22^. Relative expression levels were calculated on the basis of serial dilutions of cDNA (125–0.2 ng) which were used to generate standard curves for each gene. PCR was carried out in triplicate using 96-well optical reaction plates. The reaction conditions comprised an initial heating step at 95 °C for 5 min followed by 44 cycles of 95 °C for 10 s, 58 °C for 35 s and 72 °C for 15 s. Specificity was confirmed by product melt curve analysis over the temperature range 50–90 °C with fluorescence acquired after every 0.5 °C increase, and the fluorescence threshold values and gene expression data were calculated using BioRad CFX96™ software. Values represent the mean of 10 replicates ± SD. Amplification efficiencies were compared by plotting ΔCt values of different primer combinations in serial dilutions against the log of starting template concentrations using CFX96 software.

### Carotenoid extraction from maize endosperm

Maize endosperm was excised by removing the seed coat and embryo. Samples were freeze-dried before extraction and were ground to a fine powder. Carotenoids in 50–100 mg samples were extracted in 15 ml methanol:ethyl acetate (6:4 v/v) at 58 °C for 20 min. The mixture was filtered and transferred to a separation funnel before we added 15 ml hexane:diethyl ether (9:1 v/v) and agitated gently for 1 min. We then added 15 ml saturated NaCl, the aqueous phase was removed, and the organic phase was washed twice with water. The samples were dried under N_2_ at 37 °C, flushed with argon and stored at −80 °C.

### UHPLC-MS

The extracts were dissolved in 210–600 μl injection solvent, which was three parts acetonitrile/methanol (7:3 v/v) to two parts acetone. Ultra-high-performance liquid chromatography (UHPLC) analysis was carried out at SCT-DATCEM, University of Lleida, using an Acquity Ultra Performance LC system linked to a photodiode array (PDA) 2996 detector (Waters Corp., Milford, MA, USA). Mass detection was achieved using an Acquity TQD tandem quadrupole mass spectrometer equipped with a Z-spray electrospray interface (Waters). MassLynx software v4.1 (Waters) was used to control the instruments and also for data acquisition and processing. UHPLC separations were carried out on an Acquity UPLC C18 BEH 130 Å, 1.7 μm, 2.1 × 150 mm reversed-phase column (Waters). The mobile phase consisted of solvent A = acetonitrile/methanol (7:3 v/v) and solvent B = 100% water. Carotenoids were quantified using a PDA detector and identified as previously described^23^ based on the order of elution from the column, ultraviolet and visible spectra, the spectral fine structure^24^, and mass fragments reported in the literature^25^ and determined by comparing the following authentic standards: β-carotene, lutein, β-cryptoxanthin and astaxanthin (Sigma-Aldrich, St Louis, MO, USA), zeaxanthin (Fluka, Buchs SG, Switzerland), phytoene and antheraxanthin (Carotenature, Lupsingen, Switzerland). Mass spectrometry (MS) analysis was carried out by atmospheric pressure chemical ionization (APCI) as previously described^25^.

## Results

### Leaf traits and their relationship with the photosynthetic rate

**Figure 1:**
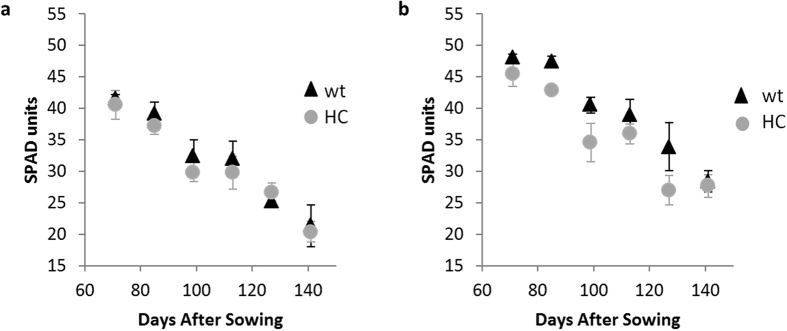
Chlorophyll content in the last fully expanded leaf during development under (**a**) N_0_ and (**b**) N_200_ treatments (values are means ± SD, n = 4) for wild-type M37W (wt, triangles) and and transgenic Carolight® corn (HC, circles). N_0_ = 0 kg ha^−1^ of N; N_200_ = 200 kg ha^−1^ of N.

The chlorophyll content of leaves (i) declined during plant growth from 43.8 ± 2.7 SPAD units at 70 DAS to 26.1 ± 2.0 SPAD units at 141 DAS and (ii) was slightly higher under the N_200_ treatment than the N_0_ treatment (Fig. 1). No consistent differences in chlorophyll level ranges were observed between the wild-type and transgenic corn lines (Fig. 1).

**Figure 2:**
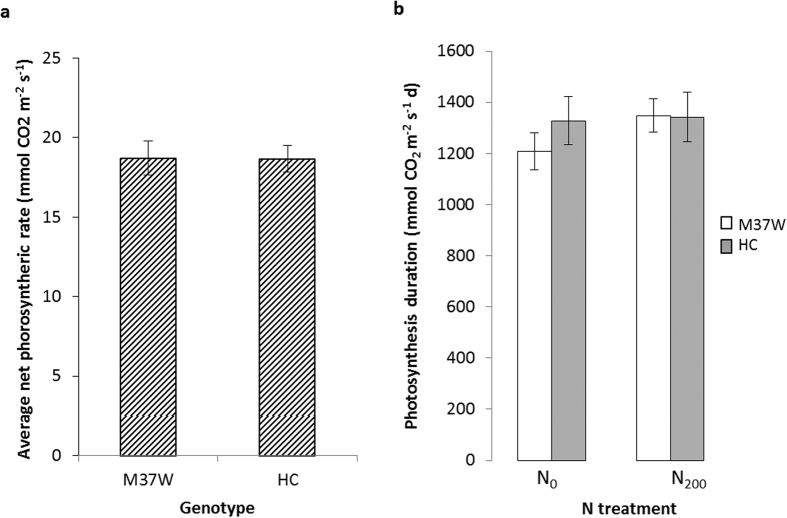
(**a**) Photosynthetic rate averaged from V_8_ (eight fully expanded leaves) to R_6_ (maturity) and for N levels for the two genotypes (M37W and Carolight®). (**b**) Duration of photosynthesis (integral of area under the curve of photosynthetic rate vs time) from V_8_ to R_6_ for the two genotypes under N_0_ (0 kg ha^−1^ of N) and N_200_ (200 kg ha^−1^ of N) treatments. Data presented as means ± SD (n = 4).

A similar decrease during growth was observed for the photosynthetic rate, with the mean value of both genotypes and treatments starting from 27.0 ± 0.9 mmol CO_2_ m^−2^ s^−1^ at V_8_ and slowly declining to 13.7 ± 0.4 mmol CO_2_ m^−2^ s^−1^ at maturity, but soil N availability did not affect the leaf photosynthetic rate. Both genotypes exhibited similar photosynthetic rates across treatments and throughout development. Consequently, the photosynthetic rate averaged across time and N levels was almost identical for both genotypes (Fig. 2a), and the duration of photosynthesis (an integral of photosynthesis over time) did not significantly differ between the genotypes under either of the N treatments (Fig. 2b).

LAI values were significantly higher in Carolight® corn than wild-type M37W at stage V_8_ under the N_0_ treatment (1.5 ± 0.1 for M37W *vs* 2.4 ± 0.3 for Carolight®) but there were no genotype-specific differences at either developmental stage under the N_200_ treatment (Table 1). The SLN was similar in both genotypes at V_8_ but was significantly higher under the N_200_ treatment. The means for the two genotypes were 2.3 ± 0.1 g m^−2^ and 3.9 ± 0.3 g m^−2^ under the N_0_ and N_200_ treatments, respectively. At silking, there were no statistically significant differences between the genotypes or treatments. The SLA was similar regardless of the genotype or treatment (Table 1).

### Biomass accumulation, grain yield and their components

**Figure 3:**
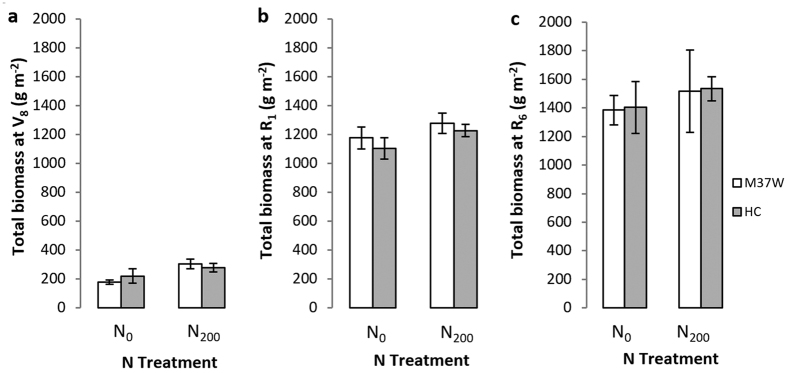
Biomass accumulation at (**a**) V_8_ (eight fully expanded leaves), (**b**) R_1_ (silking) and (**c**) R_6_ (maturity) for the two genotypes (M37W and Carolight®). Values are means ± SD, n = 4. N_0_ = 0 kg ha^−1^ of N (urea); N_200_ = 200 kg ha^−1^ of N (urea).

Biomass increased from V_8_ to R_1_ and from R_1_ to R_6_, but surprisingly the responses to N fertilization were negligible (Fig. 3). There were no statistically significant differences in total biomass accumulation between the two genotypes (Fig. 3), and similar values were observed when analyzing the biomass partition between stems, leaves and cobs in both genotypes under the N_0_ and N_200_ treatments.

**Figure 4:**
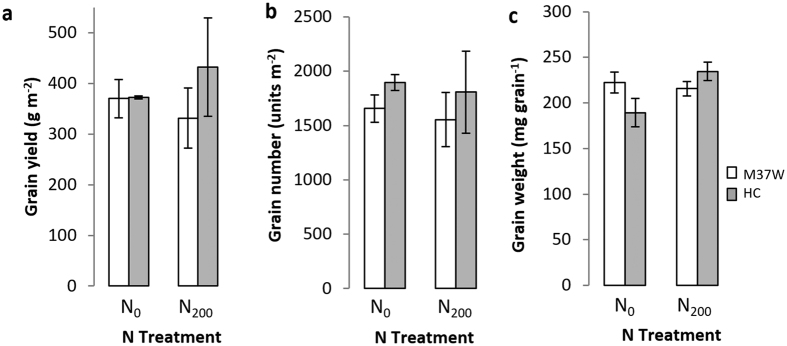
(**a**) Grain yield, (**b**) grain number and (**c**) individual grain weight for the two genotypes (M37W and Carolight®). Values are means ± SD, n = 4. N_0_ = 0 kg ha^−1^ of N (urea); N_200_ = 200 kg ha^−1^ of N (urea).

Grain yield was similar in both genotypes under both N treatments (Fig. 4a), even though Carolight® yield tended to be higher than the wild type under N fertilization. The grain yield was more closely related to grain number (R^2^ = 0.85, P < 0.05) than to average grain weight (R^2^ = 0.34, NS). There was no significant difference in grain number between genotypes, but there was a tendency towards a higher grain number in Carolight® than wild-type corn: 1895 ± 247 vs 1656 ± 126 grains m^−2^ under the N_0_ treatment and 1807 ± 378 vs 1554 ± 72 grains m^−2^ under the N_200_ treatment (Fig. 4b). There was no significant difference between the genotypes in terms of average grain weight, and no consistent trends either (Fig. 4c).

### Nitrogen content and economy

Nitrogen uptake was similar for the Carolight® and M37W plants under both N treatments (Table 2). NHI and NUtE, which were reduced by fertilization, were similar for both genotypes, and the similar levels of N partitioning and utilization efficiency were consistent regardless of the N treatment (Table 2).

### Source/sink relationship

**Figure 5:**
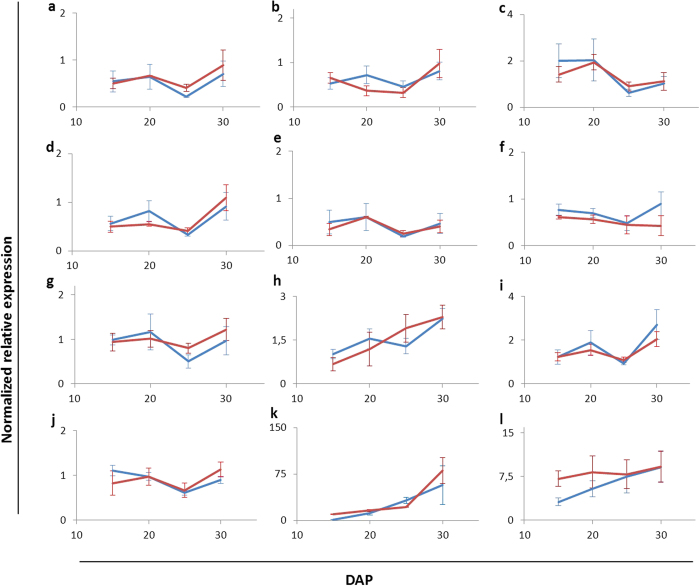
Quantitative real-time RT-PCR analysis of endogenous carotenogenic genes and transgenes in Carolight® plants grown in the field (HC-F) or in the greenhouse (HC-GH). Data show relative mRNA levels in the immature endosperm of HC-F and HC-GH plants at four developmental stages (15, 20, 25 and 30 DAP) normalized against corn *actin* mRNA and presented as the mean of 10 biological replicates. (**a**) *Zmbch1*, β-carotene hydroxylase 1; (**b**) *Zmbch2*, β-carotene hydroxylase 2; (**c**) *Zmcrtiso,* carotene isomerase; (**d**) *Zmcyp97a*, carotene ε-hydroxylase; (**e**) *Zmcyp97b*; (**f**) *Zmcyp97c*; (**g**) *Zmlycb*, lycopene β-cyclase; (**h**) *Zmlyce*, lycopene ε-cyclase; (**i**) *Zmpds*, phytoene desaturase; (**j**) *Zmzds*, ζ-carotene desaturase; (**k**) *Pacrti*, phytoene desaturase; (**l**) *Zmpsy1,* phytoene synthase*. Zm, Zea mays; Pa, Pantoea ananatis.*

At maturity, the average grain weight was consistently higher in the fully-leaved plants compared to plants that had been defoliated, with the exception of two leaves adjacent to the ear (Fig. 5). The average response to defoliation was similar in both genotypes: the grain weight was reduced by 31 ± 4% in M37W and by 28 ± 5% in Carolight® (Supplementary Fig. 1). Interestingly, in both genotypes, the grains from the apical part of the cob (the smallest grains) were the most affected: the weight of apical grains was reduced by 41 ± 9% due to defoliation whereas the weight of the central and basal grains was reduced by only 26 ± 8% (Supplementary Fig. 1).

In fully-leaved plants, the percentage of N in the grains was similar in both genotypes and consistently lower (1.87 ± 0.01%) than in the defoliated plants (2.05 ± 0.01%). This may have occurred because defoliation also reduced the grain number by ~25% in both genotypes.

### Expression of *Zmpsy1* and *Pacrt*I transgenes in greenhouse and field-grown plants

*Zmpsy1* and *Pacrt*I expression levels in the endosperm of plants grown in the field or in the greenhouse were measured by quantitative real-time RT-PCR at four time points, corresponding to four different endosperm developmental stages during grain filling: 15, 20, 25 and 30 days after pollination (DAP). Both transgenes were expressed in both environments, and in both cases *Pacrt*I mRNA accumulated at three-fold lower levels than *Zmpsy1* mRNA at all time points. The expression profile of *Pacrt*I was similar under field and greenhouse conditions (Fig. 5k). There was a two-fold increase in *Zmpsy1* mRNA levels in young endosperm (15 DAP) for plants growing in the greenhouse compared to plants in the field, but the expression levels in the two environments converged during seed development (Fig. 5l).

### Endogenous carotenogenic gene expression in the transgenic plants

We measured the accumulation of transcripts representing 10 endogenous carotenogenic genes in the developing endosperm of transgenic plants in both environments: β-carotene hydroxylase 1 (*Zmbch1*), β-carotene hydroxylase 2 (*Zmbch2*), carotene isomerase (*Zmcrtiso*), carotene ε-hydroxylase (*Zmcyp97a*, *Zmcyp97b*, and *Zmcyp97c*), lycopene β-cyclase (*Zmlycb*), lycopene ε-cyclase (*Zmlyce*), phytoene desaturase (*Zmpds*) and ζ-carotene desaturase (*Zmzds*). No significant differences in the expression levels of these endogenous carotenogenic genes were observed between the two environments, and the abundance of each transcript did not change substantially over time (Fig. 5a–j). *Zmbch2* mRNA was more than 1000-fold more abundant than *Zmbch1* mRNA, as previously reported^3^. *Zmcyp97A* and *Zmcyp97B* mRNA accumulated to high levels in both environments and were more than 100-fold more abundant than *Zmcyp97C* mRNA. *Zmlycb* mRNA was more abundant than *Zmlyce* mRNA.

### Carotenoid accumulation during endosperm development

**Figure 6:**
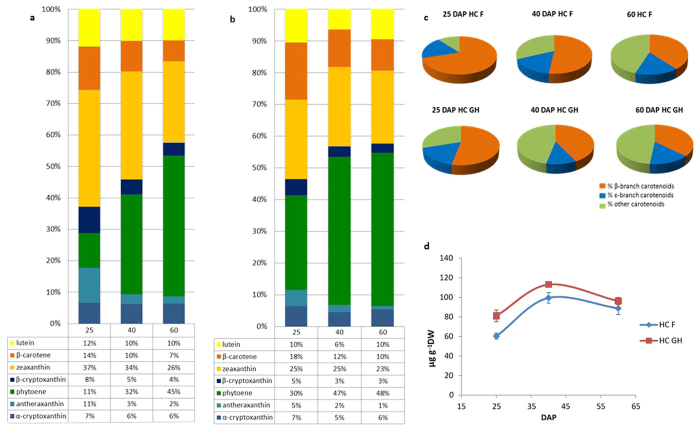
Carotenoid content and composition in the endosperm of Carolight® plants grown in the field (HC-F) or in the greenhouse (HC-GH). Individual carotenoids (%) in the 25, 40 and 60 DAP endosperm of (**a**) HC-F plants and (**b**) HC-GH plants. (**c**) Distribution of β and ε branch carotenoids in HC-F and HC-GH endosperm at 25, 40 and 60 DAP. (**d**) Total carotenoid content of HC-F and HC-GH endosperm at 25, 40 and 60 DAP.

We measured the carotenoid content and composition in the endosperm of the plants in both environments at three developmental stages (25, 40 and 60 DAP). The prevalent carotenoids were phytoene, zeaxanthin, lutein, β-carotene, α-cryptoxanthin, β-cryptoxanthin and antheraxanthin. The maximum total carotenoid accumulation occurred at ~40 DAP in both environments, and was 113 ± 7.5 μg/g DW for plants in the greenhouse compared to 99 ± 2.7 μg/g DW for plants in the field. However, no significant differences in the total carotenoid content were observed in the mature kernels at 60 DAP. The total carotenoid content was 96.8 ± 3.7 μg g^−1^ DW for plants in the greenhouse compared to 88.7 ± 6.5 μg g^−1^ DW for plants grown in the field (Fig. 6d). M37W plants under the same conditions accumulated only minimal amounts of lutein, zeaxanthin and antheraxanthin in both environments, consistent with earlier reports^21^.

The levels of each carotenoid varied during endosperm development, with significant differences in their relative proportions when comparing plants in the greenhouse and in the field. However, these differences became less pronounced during further development and plants in both environments contained similar levels of each carotenoid at maturity (Fig. 6a and 6b). This was confirmed by measuring the distribution of carotenoids representing the β and ε branches of the carotenoid pathway (Fig. 6c). Young endosperm tissue (25 DAP) accumulated a higher proportion of β branch carotenoids in the field (70% of total carotenoids) than the greenhouse (53% of total carotenoids) (Fig. 6a and 6b) perhaps reflecting the higher rate of conversion of β-carotene into downstream products such as zeaxanthin (37% for field plants *vs* 25% for greenhouse plants), antheraxanthin (11% *vs* 5%) and β-cryptoxanthin (8% *vs* 5%). Indeed, higher proportions of these carotenoids were detected in field-grown plants compared to their counterparts in the greenhouse, with corresponding lower amounts of β-carotene (Fig. 6a and 6b). We measured a gradual decline in the accumulation of β-branch carotenoids in the endosperm of field-grown plants over time, compensated by a concomitant increase in the levels of early carotenoids up to lycopene. This resulted in a similar carotenoid profile in mature endosperm (60 DAP) in both environments. The proportion of β-branch carotenoids was higher than the proportion of ε-branch carotenoids at all time points. The proportion of total carotenoids in the ɛ-branch (α-cryptoxanthin and lutein) remained constant during seed development (Fig. 6c).

Phytoene was the most abundant carotenoid in the endosperm of plants grown in both environments. The levels of phytoene increased during endosperm maturation under both conditions, representing ~50% of total carotenoids at maturity (Fig. 6a and 6b). Plants in the greenhouse accumulated more β-carotene (~14.6 ± 1.85 μg g^−1^ DW, equivalent to 10% of total carotenoids) than field-grown plants (~9.60 ± 1.21 μg g^−1^ DW, equivalent to 7% of total carotenoids) (Fig. 6a and 6b).

## Discussion

### Grain yield, biomass accumulation and partitioning

Novel crops with enhanced traits should be tested against their near-isogenic wild-type counterparts under a variety of environmental conditions to ensure that performance is maintained in the environments that are likely to be most relevant for commercial deployment. However, it is rare for such comprehensive analysis to be performed even for the simplest first-generation input traits such as herbicide tolerance and pest resistance, and unprecedented for second-generation output traits such as enhanced metabolic pathways. This is particularly the case for products with humanitarian applications developed outside the private sector. We therefore addressed the hypothesis that a nutritionally enhanced crop such as Carolight® should be similar in terms of biomass and yield to its near isogenic line M37W, and that carotenoid production should be stable under a variety of conditions and environments.

During early growth (V_8_) without fertilizer treatment (N_0_), the LAI of Carolight® plants was significantly higher than that of M37W plants, but by silking (R_1_) no significant differences were detected between the genotypes. The early differences in LAI between genotypes were probably not substantial enough to cause statistically significant differences in radiation interception, the rate of photosynthesis or the duration of photosynthesis (Fig. 2). Furthermore, there were no differences between the two genotypes in terms of plant biomass at the three developmental stages we investigated (Fig. 3). These results indicate that the plant biomass, as well as height and other morphological characteristics were similar in each genotype. Very few experiments have been reported comparing transgenic maize lines and their non-transgenic counterparts in terms of biomass and partitioning under field conditions^11^,^26^. A higher grain yield was observed for Bt hybrids compared to near-isogenic lines in the absence of lepidopteran pests, which was attributed to greater dry matter production in the leaves and grains of the Bt hybrids while the harvest index and leaf chlorophyll content did not differ significantly between the genotypes^11^. More recent comparisons between other Bt maize hybrids and their near-isogenic counterparts revealed similar grain numbers per plant and overall grain yields^26^.

When N fertilizer was provided in our experiments (N_200_) most of the measured traits remained at similar levels compared to the N_0_ treatment (Table 2, Figs 1, 2 and 3) or became higher, e.g. the grain yield (Table 1, Fig. 4). The response to N availability may vary under different agronomic environments depending on the genotype and environmental conditions^27^. Our experiments were designed to determine whether the transgenic and non-transgenic plants responded differently to changes in the N supply. The response to N was generally similar in both genotypes, indicating that the introduced transgenes do not influence the manner in which M37W corn plants respond to N availability. This is in agreement with earlier comparisons of Bt hybrids and near-isogenic counterparts, in which the N levels at V_7_, R_1_ and R_6_ were similar in both genotypes^11^. Our data therefore suggest that Carolight® plants will perform well under field conditions and that the enhanced metabolic phenotype does not attract a yield penalty.

Changing the source-sink relationship by defoliation at silking reduced the grain weight by ~30% in both genotypes, and there was no significant difference between them (Supplementary Fig. 1). The response was strongest in the grains in the apical third of the ear which are always smaller than those of the basal and central thirds because growth starts 4–5 days later than the basal grains and there is a greater likelihood of abortion^28^. For this reason, the apical grain weight reduction we observed in both the Carolight® and M37W plants was probably due to the severity of source limitation for apical grain growth. Importantly, the additional metabolic requirements of Carolight® endosperm did not increase the sensitivity to source limitation compared to the wild-type variety, suggesting that sensitivity to abiotic stress (impairing the balance of resources between grain growth and assimilate availability) is not increased by the introgression of carotenogenic transgenes. The reduction in grain size and number in response to defoliation at silking (before grain set) agrees with earlier reports of significant decreases in grain yield caused by defoliation at this developmental stage^17^. Grain number is mainly determined during the critical 30-day period bracketing silking^29^, which is characterized by the growth of the juvenile ear, containing the female florets^30^. The abortion process then affects a proportion of the pollinated florets. Grain weight potential is largely determined during the same period^31^ but final grain weight is realised during the effective period of grain filling^17^. The relative N content in the grains was similar in both genotypes, and increased significantly (~10%) in both varieties following defoliation. N provision to growing grains can be restricted at source by limiting either its availability from post-anthesis assimilation or its translocation from leaves^32^. Grain growth in cereals therefore tends to be sink-limited whereas N accumulation in grains is usually source-limited^33^. Defoliation is therefore expected to result in a lower proportional incorporation of N^17^. However, defoliation also reduced the grain number in our study, and this may explain the higher rate of N incorporation we observed in both genotypes.

### Comparison of the introduced trait under controlled and field environments

As well as comparing the performance of novel crops under diverse field conditions, it is also important to identify differences between plants grown in the greenhouse and field given that transgenic varieties must be grown in greenhouses during the first stages of product development and field testing is only possible with the appropriate permits from national competent authorities^34^. The total carotenoid content was similar in greenhouse and field plants, reaching a peak of *~*106 ± 7 μg g^−1^ DW at 40 DAP, which is significantly higher than the maximum of 86 ± 0.65 μg g^−1^ DW previously reported for a T1 Carolight® line^21^. The capacity for carotenoid accumulation in Carolight® corn therefore appears to increase by nearly 20% between generations T1 and T12. Similarly, a 7–20-fold increase in the accumulation of a recombinant protein expressed in transgenic corn endosperm was reported between generations T1 and T6^35^. Our results therefore confirm that the expression levels of one or more introduced transgenes may be increased through a selection and breeding program. This conclusion is important because it provides an early “selectable trait” to aim for in commercial breeding programs.

We observed a 25% higher total carotenoid content in the young endosperm of greenhouse plants compared to field plants, but this gap had closed by maturity (60 DAP) with plants in both environments accumulating ~95 μg g^−1^ DW total carotenoids in the endosperm. Studies on the effects of cultivation practices and conditions (such as field vs greenhouse) are limited in cereals, and have been reported mainly in other crops such as tomato^36^,^37^ and pepper^38^,^39^,^40^. A greenhouse study in tomato found that β-carotene, lycopene and lutein levels were negatively affected by light intensity^36^. Brandt *et al*.^37^ detected higher lycopene levels in tomato fruits grown in a greenhouse compared to those grown in the field. Thirteen greenhouse-grown varieties of *Capsicum annuum* and *C. chinense* produced more carotenoids than field-grown plants, reflecting the 20% higher light intensity in the field compared to the greenhouse^38^. Cultivar-specific responses were measured among red pepper cultivars but the majority produced more carotenoids in the greenhouse than the field^39^. The carotenoid content of three other *C. annuum* cultivars has also been shown to decline by up to three-fold in the field compared to the greenhouse^40^. Light intensity can vary substantially in a field or in a greenhouse, thus possibly affecting carotenogenic gene expression and/or carotenoid production. Corn is cultivated in fields during summer, when the light intensity can reach extreme values. In our region, the temperature can reach 40 °C in August, which is when Carolight® reaches its reproductive stage, whereas the temperature of the greenhouse did not exceed 28 °C. This may explain the relatively low levels of carotenoid production in the Carolight® plants growing in the field compared to the greenhouse during the first period of endosperm development, whereas the lower field temperatures later in development allowed the field-grown plants to catch up with their greenhouse-dwelling counterparts.

When we analyzed the carotenoid profile, the proportions of the major carotenoids found in the endosperm varied during development between the plants in the greenhouse and field, although again these differences had disappeared by maturity and the profiles in mature kernels were similar regardless of where the plants were cultivated. The young endosperm tissue of field plants contained ~20% higher levels of β-branch carotenoids than greenhouse plants, but this reflected the specific accumulation of more zeaxanthin, β-cryptoxanthin and antheraxanthin and not β-carotene. Indeed, β-carotene was only ~3% more abundant in the greenhouse plants, consistent with the negative response of β-carotene to light intensity in tomato^36^. Higher temperatures and increased exposure to solar radiation were shown to reduce β-carotene accumulation in the fruits of the same tomato cultivar^41^. These data suggest that the high temperature and light intensity in the field during summer growth may negatively affect the β-carotene content of Carolight® corn. The proportion of ε-branch carotenoids remained constant in both environments during development (25–60 DAP) which suggests that differences in β-branch carotenoids are compensated by changes in the levels of phytoene, the precursor of downstream metabolites in the pathway. In both environments, the phytoene content increased over time, from young to mature endosperm, consistent with a gradual decline in the level of β-branch carotenoids. Phytoene gradual accumulation probably reflects a metabolic bottleneck at the level of *Pacrt*I (phytoene desaturase), which converts phytoene into lycopene. This finding is important because it suggests that the performance of *Pacrt*I might not be optimal, and this could be remedied in subsequent Carolight® products, particularly with the rapid development of genome editing technologies^42^,^43^. The transient differences in β-branch carotenoids described above are likely to reflect the abundance and/or activity of the enzymes acting downstream of phytoene and they also suggest an additional level of control of transgene expression by the environment. Carotenoid accumulation in seeds, fruits and flowers correlates with the abundance of transcripts representing key carotenogenic genes^1^,^44^. Therefore, quantitative real-time RT-PCR was used to determine whether the bottleneck could be explained by the low abundance of *Pacrt*I mRNA, and whether the transient differences in β-branch carotenoids also reflected differences at the mRNA level. In both environments and at all time points, *Pacrt*I mRNA accumulated in the endosperm at significantly lower levels (~3-fold lower) than *Zmpsy1* mRNA. We also found that greenhouse plants accumulated twice as much *Zmpsy1* mRNA in the young endosperm (15 DAP) than field plants which partially explains the transient differences in carotenoid profiles we observed. This is consistent with the significant difference in the total carotenoid content of the endosperm of greenhouse and field plants at 25 DAP, suggesting that *Zmpsy1* mRNA accumulates at higher levels in the endosperm of greenhouse plants at ~15 DAP thus increasing the flux in the downstream carotenoid pathway. *Zmpsy1* mRNA levels then converged in the greenhouse and field plants between 20 and 30 DAP. Similarly, a correlation between *psy1* expression and total carotenoid levels was specifically observed at 20 DAP^45^. The differential expression of *Zmpsy1* mRNA alone while other endogenous or exogenous carotenogenic transcripts were expressed at consistent levels suggests that *Zmpsy1* mRNA and/or its promoter is peculiarly sensitive to the different environmental conditions in the greenhouse and field. These environments are characterized by differences in light quality and temperature, as well as biotic and abiotic stresses, and this could influence carotenogenic gene/transgene expression or carotenoid accumulation. Light regulates genes and gene products related to photosynthesis, including carotenoids^46^. The rice genes *Ospsy1* and *Ospsy2* contain *cis*-acting elements involved in light regulation^47^ as are the three equivalent corn genes, *Zmpsy1-3*^48^,^49^. It is unclear whether the three *psy* genes in corn have overlapping functions in the modulation of carotenoid synthesis in different tissues and in response to developmental and/or stress signals. In corn leaves, carotenogenesis may require both phytochrome-dependent and phytochrome-independent photoregulation of *psy2* as well as non-photoregulation of *psy1*^45^. Non-photoregulated PSY1 is the main enzyme responsible for carotenoid synthesis in corn endosperm^50^ whereas PSY3 mediates carotenoid synthesis in the roots induced by abiotic stress^49^. PSY1 is required for heat stress-induced carotenoid biosynthesis in corn to protect plastid membranes in photosynthetic tissues^51^,^52^. However, although there is a correlation between abiotic stress and *Zmpsy2* mRNA levels in the leaves and *Zmpsy3* mRNA levels in the roots, *Zmpsy1* mRNA levels do not appear to respond to abiotic stress at all. Importantly, these studies were performed under laboratory conditions and there are no studies thus far reporting the response of *Zmpsy1* to the combined stresses that may be encountered under field conditions. Interestingly, we controlled the *Zmpsy1* transgene using the wheat low-molecular-weight glutenin promoter, which is not known to be influenced by light or abiotic stress. Our study therefore provides the first evidence that environmental factors can influence either this promoter or the *Zmpsy1* gene.

Although we did not observe any major differences between greenhouse and field plants in the expression of most endogenous carotenogenic genes during endosperm development (15–30 DAP), *Zmlycb* mRNA was four times more abundant than *Zmlyce* mRNA in both environments, which is important because the corresponding enzymes compete to divert flux into the β-branch and ε-branch^53^. This expression profile could therefore explain the higher content of β-branch carotenoids (~30%) at maturity compared to ε-branch carotenoids (~10%) in both greenhouse and field plants. However, the differential expression of hydroxylase genes may also influence the carotenoid profile. Two classes of enzymes catalyze the hydroxylation of α and β ionone rings in higher plants: CYP97-type heme-containing cytochrome P450 hydroxylases^54^,^55^ and ferredoxin-dependent β-carotene hydroxylase (BCH)-type non-heme di-iron hydroxylases^56^,^57^,^58^. BCH-type enzymes typically show limited activity towards the ε-ring of α-carotene but significant activity towards the β-ring, although *Zmbch1* also promotes the accumulation of α-carotene^59^. CYP97A appears to be responsible for catalyzing the hydroxylation of the β-ring of α-carotene whereas CYP97C is mainly active against the ε-ring^55^,^60^. The enzyme PuCHY1, a CYP97B produced by the red alga *Porphyra umbilicalis*, is also responsible for β-ring hydroxylation^61^. We found that *Zmbch2* mRNA accumulated to levels 100-fold greater than *Zmbch1* mRNA and that *Zmcyp97a* and *Zmcyp97b* were expressed at levels at least 10-fold higher than *Zmcyp97c* in the endosperm of both the greenhouse and field plants. The higher content of β-branch carotenoids in the Carolight® endosperm in both environments can therefore be explained by these specific gene expression profiles.

In conclusion, we have shown that the high-carotenoid transgenic corn line Carolight® does not behave in a significantly different manner, in terms of phenology and agronomic performance, to its wild-type counterpart. Importantly, we tested for differences under diverse environmental conditions to confirm that the engineered variety is likely to perform well in a range of environments relevant to commercial deployment. To our knowledge, this is the first time that a transgenic variety with a second-generation output trait has been characterized in such a comprehensive manner although such comparisons are highly recommended during the development of novel crops to avoid disappointing field performance and late-stage product development failure. Minor differences in parameters such as endosperm carotenoid composition and LAI were measured in the early stages of development, but they did not affect the grain yield or any other growth/development phenotypes. Importantly we provide a molecular basis for this behavior which might help the development of improved versions not only of Carolight® but also other second-generation and third-generation crops. Carolight® also behaves in a similar manner in a controlled environment and under field conditions, which is an essential requirement to support further product development given that early studies are restricted to the greenhouse under the current regulatory framework and field studies are only permitted with permission from national competent bodies. The transient minor differences we observed appear to reflect differences in the expression of a single gene (*Zmpsy1*) thus showing that even relatively minor differences in the regulation of transgene expression can affect the behavior of engineered crop plants in a significant and environmental-dependent manner.

## Additional Information

**How to cite this article**: Zanga, D. *et al*. A carotenogenic mini-pathway introduced into white corn does not affect development or agronomic performance. *Sci. Rep.* **6**, 38288; doi: 10.1038/srep38288 (2016).

**Publisher's note:** Springer Nature remains neutral with regard to jurisdictional claims in published maps and institutional affiliations.

## Supplementary Material

Supplementary Figure 1

## Figures and Tables

**Table 1:** 

Stage of development	N treatment	Genotype	LAI	SLA (cm^2^ g^−1^)	SLN (mg N cm^−2^)
V_8_	N_0_	M37W	1.5 ± 0.1	208.3 ± 7.4	2.2 ± 0.4
		Carolight®	2.4 ± 0.3	216.9 ± 7.1	2.5 ± 0.6
	N_200_	M37W	2.7 ± 0.3	201.6 ± 3.2	4.2 ± 0.4
		Carolight®	2.5 ± 0.1	201.5 ± 6.9	3.7 ± 0.7
R_1_	N_0_	M37W	4.5 ± 0.2	160.4 ± 9.4	2.2 ± 0.1
		Carolight®	4.6 ± 0.4	151.7 ± 8.5	2.8 ± 0.4
	N_200_	M37W	4.6 ± 0.3	148.8 ± 7.4	3.2 ± 0.3
		Carolight®	5.6 ± 1.2	157.1 ± 3.1	2.3 ± 0.4

Leaf traits of wild-type M37W and transgenic Carolight® corn grown in the field (values are means ± SD, n = 4) with no fertilizer (N_0_) or fertilized with 200 kg ha^−1^ of N (N_200_) at V_8_ (eight fully expanded leaves) and R_1_ (silking). LAI = leaf area index, SLA = specific leaf area, and SLN = specific leaf nitrogen.

**Table 2:** 

N treatment	Genotypes	NHI (%)	NUtE (g_grain_ g_N_^−1^)	N Uptake (g_N_ m^−2^)
N_0_	M37W	54.6 ± 1.1	29.0 ± 0.9	12.7 ± 1.0
	Carolight®	56.0 ± 5.1	29.7 ± 2.7	12.7 ± 2.21
N_200_	M37W	38.7. ± 3.1	20.4 ± 0.9	16.4 ± 0.87
	Carolight®	45.1 ± 8.7	24.3 ± 4.8	17.3 ± 1.0

Nitrogen harvest index (NHI), nitrogen utilization efficiency (NUtE) and total nitrogen uptake at maturity for wild-type M37W and transgenic Carolight® corn under two N treatments (values are means ± SD, n = 4). N_0_ = 0 kg ha^−1^ of N (urea); N_200_ = 200 kg ha^−1^ of N (urea).

## References

[b1] Zhu C. . The regulation of carotenoid pigmentation in flowers. Arch. Biochem. Biophys. 504, 132–141 (2010). 20688043 10.1016/j.abb.2010.07.028

[b2] Havaux M. & Niyogi K. K. The violaxanthin cycle protects plants from photo-oxidative damage by more than one mechanism. Proc Natl Acad Sci USA 96, 8762–8767 (1999). 10411949 10.1073/pnas.96.15.8762 PMC17590

[b3] Li Y. & Walton C. Violaxanthin Is an Abscisic Acid Precursor in Water-Stressed Dark-Grown Bean Leaves. Plant Physiol. 92, 551–559 (1990). 16667314 10.1104/pp.92.3.551 PMC1062333

[b4] Lindgren L. O., Stålberg K. G. & Höglund A. S. Seed-specific overexpression of an endogenous Arabidopsis phytoene synthase gene results in delayed germination and increased levels of carotenoids, chlorophyll, and abscisic acid. Plant Physiol. 132, 779–785 (2003). 12805607 10.1104/pp.102.017053 PMC167017

[b5] Omoni O. A. & Aluko E. R. The anti-carcinogenic and anti-atherogenic effects of lycopene: a review. Trends Food Sci Technol. 16, 344–350 (2005).

[b6] Paine J. . Improving the nutritional value of Golden Rice through increased pro-vitamin A content. Nat Biotechnol. 23, 482–487 (2005). 15793573 10.1038/nbt1082

[b7] Zhu C. . Combinatorial genetic transformation generates a library of metabolic phenotypes for the carotenoid pathway in maize. Proc Natl Acad Sci USA 105, 18232–18237 (2008). 19011084 10.1073/pnas.0809737105 PMC2587607

[b8] Farré G. . Nutritious crops producing multiple carotenoids - a metabolic balancing act. Trends Plant Sci. 16, 532–540 (2011). 21900035 10.1016/j.tplants.2011.08.001

[b9] Sanahuja G. . A question of balance - achieving appropriate nutrient levels in biofortified staple crops. Nutr Res Rev. 26, 235–245 (2013). 24134863 10.1017/S0954422413000176

[b10] Zhu C. . Biofortification of plants with altered antioxidant content and composition: genetic engineering strategies. Plant Biotechnol. J. 11, 129–141 (2013). 22970850 10.1111/j.1467-7652.2012.00740.x

[b11] Subedi K. D. & Ma B. L. Dry matter and nitrogen partitioning patterns in Bt and non-Bt near-isoline maize hybrids. Crop Sci. 47, 1186–1192 (2007).

[b12] Elmore R. W. . Glyphosate-resistant soybean cultivar response to glyphosate. Agron J. 93, 404–407 (2001).

[b13] Elmore R. W. . Glyphosate-resistant soybean cultivar yields compared with sister lines. Agron J. 93, 408–412 (2001).

[b14] Marra M., Piggott N. E. & Carlson G. A. The net benefits, including convenience, of Roundup Ready® soybeans: Results from a National Survey (NSF Center for IPM Technical Bulletin, 2004).

[b15] Decourcelle M. . Combined transcript, proteome, and metabolite analysis of transgenic maize seeds engineered for enhanced carotenoid synthesis reveals pleotropic effects in core metabolism. J Exp Bot. 66, 3141–3150 (2015). 25796085 10.1093/jxb/erv120 PMC4449536

[b16] Gastal F. & Lemaire G. N uptake and distribution in crops: an agronomical and ecophysiological perspective. J Exp Bot. 53, 789–799 (2002). 11912222 10.1093/jexbot/53.370.789

[b17] Borrás L., Slafer G. A. & Otegui M. E. Seed dry weight response to source-sink manipulations in wheat, maize and soybean: a quantitative reappraisal. Field Crop Res. 86, 131–146 (2004).

[b18] Seebauer J. R., Singletary G. W., Krumpelman P. M., Ruffo M. L. & Below F. E. Relationship of source and sink in determining kernel composition of maize. J Exp Bot. 61, 511–519 (2010). 19917600 10.1093/jxb/erp324 PMC2803218

[b19] Ritchie S. W., Hanway J. J. & Benson G. O. How a Corn Plant Develops. CES 21 (Iowa State Univ., Ames, IA 1993).

[b20] Abeledo L. G., Savin R. & Slafer G. A. Leaf Photosynthesis During Grain Filling Under Mediterranean Environments: Are Barley or Traditional Wheat More Efficient Than Modern Wheats? J Agro Crop Sci. 200, 172–182 (2014).

[b21] Farre´ G. . Targeted transcriptomics and metabolic profiling reveals temporal bottlenecks in the maize carotenoid pathway that can be addressed by multigene engineering. Plant J. 75, 441–455 (2013). 23607313 10.1111/tpj.12214

[b22] Naqvi S. . Synergistic metabolism in hybrid corn reveals bottlenecks in the carotenoid pathway and leads to the accumulation of extraordinary levels of the nutritionally important carotenoid zeaxanthin. Plant Biotechnol J. 9, 384–393 (2011). 20807370 10.1111/j.1467-7652.2010.00554.x

[b23] Rivera S. M. . Fast quantitative method for the analysis of carotenoids in transgenic maize. J. Agric. Food Chem. 61, 5279–5285 (2013). 23678974 10.1021/jf400694z

[b24] Britton G., Liaaen-Jenson S. & Pfander H. Carotenoids Handbook. Photosynthetica 42, 186–186 (2004).

[b25] Rivera S., Vilaró F. & Canela R. Determination of carotenoids by liquid chromatography/mass spectrometry: Effect of several dopants. Anal. Bioanal. Chem. 400, 1339–1346 (2011). 21380750 10.1007/s00216-011-4825-6

[b26] Laserna M. P., Maddonni G. A. & Lopez C. G. Phenotypic variations between non-transgenic and transgenic maize hybrids. Field Crop Res. 134, 175–184 (2012).

[b27] Cirilo A. . Morphophysiological traits associated with maize crop adaptations to environments differing in nitrogen availability. Field Crop Res. 113, 116–124 (2009).

[b28] Tollenaar M. & Daynard T. B. Kernel growth and development at two positions on the ear of maize (*Zea mays*). Can J Plant Sci. 58, 189–197 (1978).

[b29] Andrade F. H., Echarte L., Rizzalli R., Della Maggiora A. & Casanovas M. Kernel number prediction in maize under nitrogen or water stress. Crop Sci. 42, 1173–1179 (2002).

[b30] Otegui M. E. & Bonhomme R. Grain yield components in maize: I. Ear growth and kernel set. Field Crops Res. 56, 247–256 (1998).

[b31] Gambín B. L., Borrás L. & Otegui M. E. Source-sink relations and kernel weight differences in maize temperate hybrids. Field Crops Res. 95, 316–326 (2006).

[b32] Simpson R. J., Lambers H. & Dalling M. J. Nitrogen redistribution during grain growth in wheat (*Triticum aestivum* L) IV Development of a quantitative model of the translocation of nitrogen to the grain. Plant Physiol. 71, 7–14 (1983). 16662801 10.1104/pp.71.1.7 PMC1065976

[b33] Dreccer M. F., Grashoff C. & Rabbinge R. Source-sink ratio in barley (*Hordeum vulgare* L.) during grain filling: effects on senescence and grain protein concentration. Field Crops Res. 49, 269–277 (1997).

[b34] Gómez-Galera S. . Field trials and tribulations – making sense of the regulations for experimental field trials of transgenic crops in Europe. Plant Biotechnol J. 10, 511–523 (2012). 22284604 10.1111/j.1467-7652.2012.00681.x

[b35] Hood E. E. . Manipulating corn germplasm to increase recombinant protein accumulation. Plant Biotechnol J. 10, 20–30 (2012). 21627759 10.1111/j.1467-7652.2011.00627.x

[b36] Ehret D. L. . Tomato fruit antioxidants in relation to salinity and greenhouse climate. J Agric Food Chem. 61, 1138–1145 (2013). 23311953 10.1021/jf304660d

[b37] Brandt S. . Effects of growing methods and conditions on the lycopene content of tomato fruits. Acta Aliment Hung. 32, 269–278 (2003).

[b38] Lee J. J., Crosby K. M., Pike L. M., Yoo K. S. & Leskovar D. I. Impact of genetic and environmental variation on development of flavonoids and carotenoids in pepper (Capsicum spp.) Sci. Hort. 106, 341–352 (2005).

[b39] Russo V. M. & Howard L. R. Carotenoids in pungent and non-pungent peppers at various developmental stages grown in the field and glasshouse. J. Sci. Food Agr. 82, 615–624 (2002).

[b40] Keyhaninejad N., Richins R. D. & O’Connel M. A. Carotenoid Content in Field-grown versus Greenhouse-grown Peppers: Different Responses in Leaf and Fruit. Hortscience 47, 852–855 (2012).

[b41] Rosales M. A. . Antioxidant content and ascorbate metabolism in cherry tomato exocarp in relation to temperature and solar radiation. J. Sci. Food Agric. 86, 1545–1551 (2006).

[b42] Bortesi L. . Patterns of CRISPR/Cas9 activity in plants, animals and microbes. Plant Biotechnol J. 10.1111/pbi.12634 (2016). PMC5103219 27614091

[b43] Zhu C. . Characteristics of genome editing mutations in cereal crops. Trends in Plant Sci. 10.1007/s11032-016-0533-4 (2016). 27645899

[b44] Fraser P. D., Truesdale M. R., Bird C. R., Schuch W. & Bramley P. M. Carotenoid biosynthesis during tomato fruit development. Plant Physiol. 105, 405–413 (1994). 12232210 10.1104/pp.105.1.405 PMC159369

[b45] Li F., Vallabhaneni R., Yu J., Rocheford T. & Wurtzel E. T. The Maize Phytoene Synthase Gene Family: Overlapping Roles for Carotenogenesis in Endosperm, Photomorphogenesis, and Thermal Stress Tolerance. Plant Physiol. 147, 1334–1346 (2008). 18508954 10.1104/pp.108.122119 PMC2442542

[b46] Pizarro L. & Stange C. Light-dependent regulation of carotenoid biosynthesis in plants. Cien. Inv. Agr. 36, 143–162 (2009).

[b47] Dibari B. . Deciphering the genomic structure, function and evolution of carotenogenesis related phytoene synthases in grasses. BMC Genomics 13, 221 (2012). 22672222 10.1186/1471-2164-13-221 PMC3413518

[b48] Gallagher C. E., Matthews P. D., Li F. & Wurtzel E. T. Gene duplication in the carotenoid biosynthetic pathway preceded evolution of the grasses. Plant Physiol. 135, 1776–1783 (2004). 15247400 10.1104/pp.104.039818 PMC519089

[b49] Li F., Vallabhaneni R. & Wurtzel E. T. PSY3, a new member of the phytoene synthase gene family conserved in the Poaceae and regulator of abiotic stress-induced root carotenogenesis. Plant Physiol. 146, 1333–1345 (2008). 18162592 10.1104/pp.107.111120 PMC2259096

[b50] Li F., Tsfadia O. & Wurtzel E. T. The phytoene synthase gene family in the Grasses: Subfunctionalization provides tissue-specific control of carotenogenesis. Plant Signaling & Behavior 4, 208–211 (2009). 19721751 10.4161/psb.4.3.7798 PMC2652530

[b51] Davison P. A., Hunter C. N. & Horton P. Overexpression of β-carotene hydroxylase enhances stress tolerance in *Arabidopsis*. Nature 418, 203–206 (2002). 12110893 10.1038/nature00861

[b52] Havaux M., Dall’Osto L. & Bassi R. Zeaxanthin has enhanced antioxidant capacity with respect to all other xanthophylls in *Arabidopsis* leaves and functions independent of binding to PSII antennae. Plant Physiol. 145, 1506–1520 (2007). 17932304 10.1104/pp.107.108480 PMC2151694

[b53] Harjes C. E. . Natural genetic variation in lycopene ε-cyclase tapped for maize biofortification. Science 319, 330–333 (2008). 18202289 10.1126/science.1150255 PMC2933658

[b54] Tian L., Musetti V., Kim J., Magallanes-Lundback M. & DellaPenna D. The *Arabidopsis* LUT1 locus encodes a member of the cytochrome p450 family that is required for carotenoid ε-ring hydroxylation activity. Proc Natl Acad Sci USA 101, 402–407 (2004). 14709673 10.1073/pnas.2237237100 PMC314197

[b55] Kim J. & DellaPenna D. Defining the primary route for lutein synthesis in plants: the role of Arabidopsis carotenoid β-ring hydroxylase CYP97A3. Proc Natl Acad Sci USA 103, 3474–3479 (2006). 16492736 10.1073/pnas.0511207103 PMC1413914

[b56] Sun Z., Gantt E. & Cunningham F. X. Cloning and functional analysis of the β-carotene hydroxylase of *Arabidopsis thaliana*. J Biol Chem. 271, 24349–24352 (1996). 8798688 10.1074/jbc.271.40.24349

[b57] Tian L. & Dellapenna D. Characterization of a second carotenoid β-hydroxylase gene from *Arabidopsis* and its relationship to the LUT1 locus. Plant Mol Biol. 47, 379–388 (2001). 11587509 10.1023/a:1011623907959

[b58] Tian L., Magallanes-Lundback M., Musetti V. & DellaPenna D. Functional analysis of β- and ε-ring carotenoid hydroxylases in *Arabidopsis*. Plant Cell 15, 1320–1332 (2003). 12782726 10.1105/tpc.011403 PMC156369

[b59] Zhou Y. . *ZmcrtRB3* encodes a carotenoid hydroxylase that affects the accumulation of α-carotene in maize kernel. J Integr Plant Biol. 54, 260–269 (2012). 22348777 10.1111/j.1744-7909.2012.01106.x

[b60] Chang S. . Cloning and Functional Characterization of the Maize (*Zea mays* L.) Carotenoid Epsilon Hydroxylase Gene. PLoS One 10, e0128758 (2015). 26030746 10.1371/journal.pone.0128758 PMC4452274

[b61] Yang L. E. . The P450-type carotene hydroxylase PuCHY1 from Porphyra suggests the evolution of carotenoid metabolism in red algae. J Integr Plant Biol. 56, 902–915 (2014). 24942088 10.1111/jipb.12229

