# Supplementary material for: A carotenogenic mini-pathway introduced into white corn does not affect development or agronomic performance
Source: Sci Rep. 2016 Dec 6;6:38288. doi: 10.1038/srep38288 (PMC5138849; doi:10.1038/srep38288)
Supplement: Supplementary Figure 1 [file srep38288-s1.pdf]

A carotenogenic mini-pathway introduced into white corn does not affect development or agronomic performance

Daniela Zanga[1], Teresa Capell[1], Gustavo A. Slafer[1,2], Paul Christou[1,2], Roxana Savin[1]

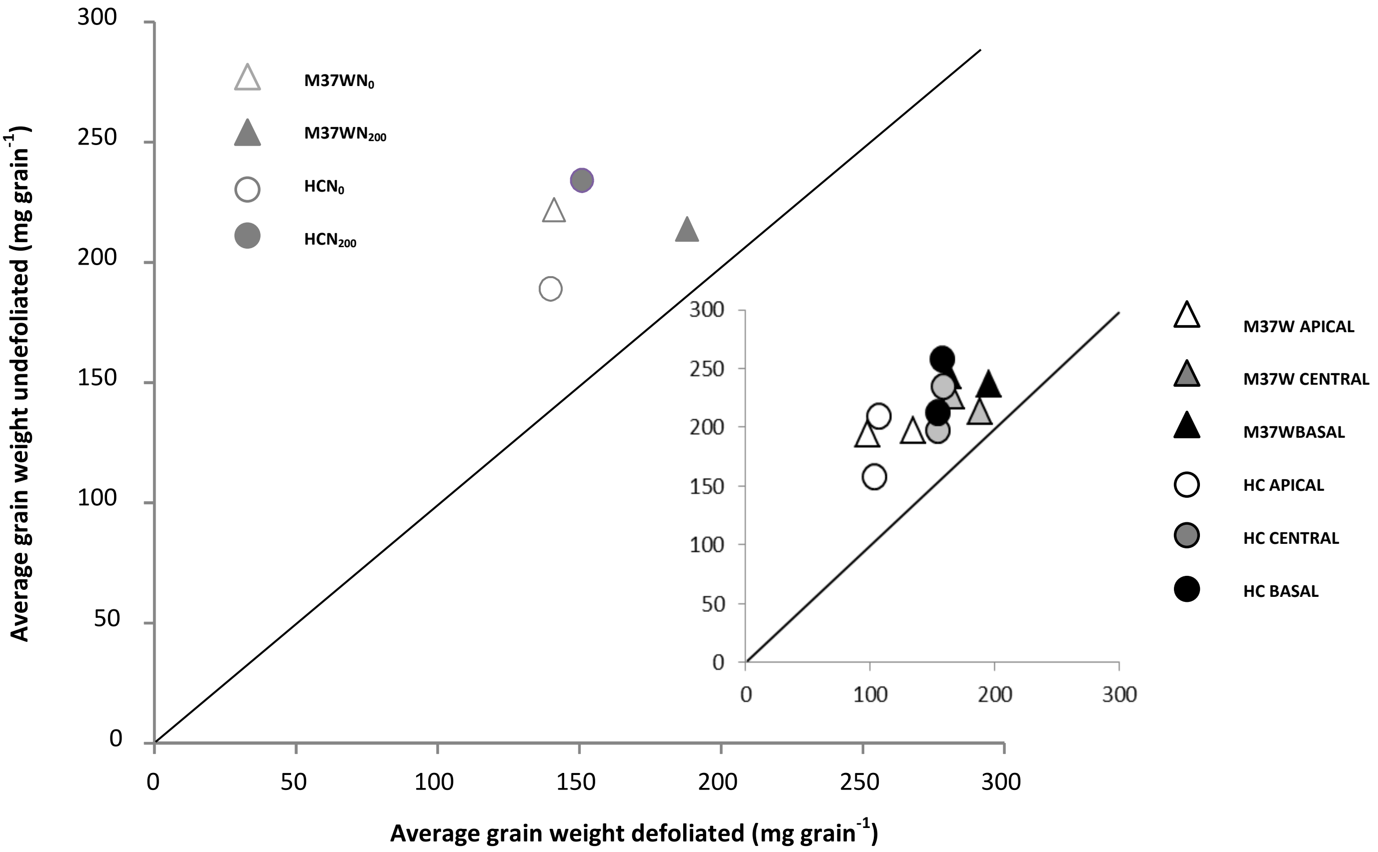

Supplementary Figure 1: Average grain weight in fully-leaved plants plotted against the corresponding values in defoliated plants for M37W (triangles) and Carolight^R (circles) grown under N_0 (open symbols) and N_{200} treatments (closed symbols). The y = x line represents the 1:1 ratio. N_0 = 0 kg ha^{-1} of N; N_{200} = 200 kg ha^{-1} of N. **Inset**: grain weight in fully-leaved plants plotted against the corresponding values in defoliated M37W and Carolight^{TM} for the basal, central and apical grains.
